# Intra-Venous Injection of Milk

**Published:** 1878-06

**Authors:** 


INTRA-VENOUS INJECTION OF MILK.—Dr. Thomas gives the following as his conclusions in the use of milk as a substitute for transfusion of blood:

1. The injection of milk into the circulation in place of blood is a perfectly feasible, safe, and legitimate procedure, which enables us to avoid most of the difficulties and dangers of the latter operation.

2. In this procedure, none but milk removed from a healthy cow within a few minutes of the injection should be employed. Decomposed milk is poisonous, and should no more be used than decomposed blood.

3. A glass funnel, with a rubber tube attached to it, ending in a very small canula, is better, safer, and more attainable than a more elaborate apparatus, which is apt, in spite of all precautions, to admit air to the circulation.

4. The intra-venous injection of milk is infinitely easier than the transfusion of blood. Any one at all familiar with surgical operations may practice it without fear of great difficulty or of failure.

5. The injection of milk, like that of blood, is commonly followed by a chill, and rapid and marked rise of temperature; then all subsides, and great improvement shows itself in the patient's condition.

6. I would not limit lacteal injections to cases prostrated by hæmorrhage, but would employ it in disorders which greatly depreciate the blood, as Asiatic cholera, pernicious anæmia, typhoid fever, etc., and as a substitute for diseased blood in certain affections which immediately call for the free use of the lancet, as puerperal convulsions, etc.

7. Not more than eight ounces of milk should be injected at one operation.

8. In conclusion, I would suggest that, if milk answers, not as good, but nearly as good, a purpose as blood under these circumstances, its use will create a new era in this most interesting department of medicine. That it will answer such a purpose, I am convinced from lengthy consideration and some experience in the matter; and I would be false to my own convictions if I did not predict for "Intra-venous Lacteal Injection" a brilliant and useful future.—*New York Medical Journal.*

